# Psychotic symptoms in Cushing’s syndrome secondary to ACTH-secreting lung carcinoid tumor: report of a case

**DOI:** 10.1192/j.eurpsy.2022.437

**Published:** 2022-09-01

**Authors:** A. Llimona González, S. Oller Canet, J. Mayans

**Affiliations:** Parc de Salut Mar, Instituto De Neuropsiquiatría Y Adicciones (inad), Barcelona, Spain

**Keywords:** Cushing’s syndrome, cushing, carcinoid, glucocorticoids

## Abstract

**Introduction:**

Cushing’s syndrome is a hormonal disorder caused by chronic exposure to excess glucocorticoids, either exogenous or endogenous. The bronchial carcinoid tumor is an extremely rare origin, described in less than 1% of cases. The most frequent psychiatric symptoms are depression and anxiety, with manic and psychotic symptoms being less frequent. Psychotic symptoms are difficult to manage, as they are usually resistant to antipsychotic treatment, which is why it is considered an indication for medical treatment of Cushing’s syndrome.

**Objectives:**

To give visibility to this type of psychotic disorders of organic origin to deepen their study as well as raise awareness among professionals dedicated to clinical care with the intention of improving their prevention.

**Methods:**

A description of a clinical case is made, accompanied by a bibliographic review on psychosis of endogenous corticosteroid origin.

**Results:**

We describe the case of a 44-year-old woman who was admitted to the charge of Internal Medicine due to Cushing’s syndrome. During her admission, she presented a debut of positive psychotic symptoms, so the liaison psychiatry team followed her up. She was diagnosed with an ACTH-secreting lung carcinoid tumor.

**Conclusions:**

This entity should be taken into account in cases of atypical psychosis in patients with compatible phenotypic characteristics.

**Disclosure:**

No significant relationships.

